# Understanding the Regional Integration Process from the Perspective of Agglomeration and Urban Networks: Case Study in Central China

**DOI:** 10.3390/ijerph191912834

**Published:** 2022-10-07

**Authors:** Liang Wang, Fangfang Zhang, Yuzhu Zang, Jian Duan

**Affiliations:** 1 School of Public Policy and Management, Tsinghua University, Beijing 100084, China; 2 Collaborative Innovation Center of Urban-Rural Coordinated Development, School of Resources and Environment, Henan University of Economics and Law, Zhengzhou 450046, China; 3 School of Public Administration, China University of Geosciences, Wuhan 430074, China; 4 School of Geography and Environmental Sciences, Zhejiang Normal University, Jinhua 321004, China

**Keywords:** spatial agglomeration, network, regional integration, urban planning, China

## Abstract

Previously, urban planning approaches have tended to convert local agglomeration into network connections to advance urban development. However, is this successful experience learned from developed counties appropriate for developing countries? Scholars hold different opinions on this debate. To answer this question, we need to examine the effects of urban agglomeration in developing countries with a quantitative method. In this paper, we introduced a method of examining network connections from a geospatial perspective to explore the practice and spatial consequences of regional integration using a new concept of “coupling distance” based on metal valence bond theory. Then we applied this method to conduct an empirical case study of the urban agglomeration in the middle reaches of the Yangtze River region in China. We found that: (1) the real integration scale of the investigated urban areas was less than one-fourth the planned area, as most of interactions between cities are local, although we see the positive facilitation of urban networks on cross-provincial integration. (2) In terms of spatial consequences, the study area demonstrated phenomena of “agglomeration shadows”, “enclaves” and “inverse integration”. Specifically, these “agglomeration shadows” were all in their province’s geometric centers, which seemed to have suffered a “central position curse”. (3) Both “enclaves” and “inverse integration” call for a readjustment of government-led regional integration planning. Differently, the former has a positive attitude towards integration while the latter holds the opposite attitude. This study hopes to provide operationalizing methods and guidelines for planners and decision makers in the field of regional integration planning.

## 1. Introduction

It is indisputable that the tide of globalization does not necessarily benefit all countries. Therefore, regional integration strategies based on city units have become the most common space typologies for many countries in order to participate in global competition and the division of labor in post-globalization [1,2]. Many of countries are actively promoting regional integration plans, hoping to achieve multiplying effects through spatial agglomeration strategies (that is 1 + 1 > 2 effect) [3,4,5]. However, is regional integration really the perfect policy tool?

Although the theoretical literature about both traditional agglomeration theory and urban network theory supports the effect of agglomeration economies advocated by regional integration [6,7], the practice of regional integration and its spatial consequences have caused an unsettled debate in the real world. For example, many studies have found that the regional integration process has had a positive effect on the whole region, as well as for inner urban individuals [8,9,10,11,12,13,14]. On the other hand, some empirical studies have also found a negative impact of the process of regional integration, in which big cities succeed at the expense of surrounding small cities [11,15,16]. Other scholars propose that the impact of the integration process on cities in the same region may be spatial heterogeneity due to different development stages, city size and city categories [17,18].

The above literature shows that the process of regional integration and its spatial consequences are uncertain. In particular, most of the conclusions are supported by the experience of developed countries, so it is still debatable whether they are feasible in developing countries [19]. However, for regional integration planners or policy makers, especially for developing countries eager to enhance their national strength by implementing regional integration policies, if the influence of the regional integration process on individual cities is ignored, then the regional integration plan may not lead to a sustainable 1 + 1 > 2 effect. Instead, ineffective spatial agglomeration strategies may prove expensive, such as repeated construction and resource waste, and even widen regional gaps and cause severe social inequality on account of wrong decisions [20,21]. Given the key role of regional integration in enhancing national strength and the feedback power of spatial agglomeration strategy practice on balanced development across cities, focusing on the regional integration process and its spatial consequences in developing countries, cannot be overemphasized.

Today, most of the measurement models of regional integration influence are from the perspective of urban networks, applying the “enterprise-city” two-mode network to convert the connection of urban physical space into an internal “urban network” [22] and then use the social network analysis method for comparative analysis, which is followed by the exploration of the regional integration process and its influence. However, these models are mostly built based on the experience of developed countries, and may not be applicable to developing countries. In fact, the process of regional integration in developed countries has evolved into a stage of networking, where it is no longer dominated by spatial constraints, such as distance (proximity and separation) and division (i.e., administrative block-groups) [23,24]. In contrast, owing to the lag of socioeconomic development, the process of regional integration in most developing countries still remains in a stage of local agglomeration [17,19]. Therefore, the measuring approach based on the experience of developed countries cannot fully explain the regional integration practice and spatial consequences in developing countries. In this study, we adopted a new research method which is different from the previous studies. We proposed a measurement tool called “coupling distance” to convert network connections (including economic, social and transportation links) into local agglomeration behaviors, and then analyzed the coupling effect between individual cities (especially the interactions between peripheral cities and central city). This model takes spatial constraints as a prerequisite when considering network connections, so that people can understand the practice of regional integration and its spatial consequences in developing countries. It can provide a new path for constructing a measurement model of regional integration in developing countries.

To sum up, this paper hopes to introduce an analysis method applicable to the process and spatial consequences of regional integration in developing countries and chose typical regions in China to illustrate the problems of this topic, so as to provide empirical support for policy practice. This study chooses the urban agglomeration in the middle reaches of the Yangtze River as a typical case, which has significant policy implications. First of all, as the world’s largest developing country, China has achieved outstanding effects of agglomeration economy in regional integration policy, but there are also obvious shortcomings, e.g., regional development inequality, excessive competition and a waste of resources [2,13,25]. These experiences and lessons have important reference value to other developing countries. Secondly, the integration process of the urban agglomeration in the middle reaches of the Yangtze River is led by the government. In the context of social economic links between cities being obviously dominated by market forces, this is an opportunity to observe the conflict of wills between government and market in the process of regional integration, which provides a new path for China and other developing countries to adjust to regional integration planning in the future. Thirdly, the urban agglomeration in the middle reaches of the Yangtze River is the first inter-provincial planning strategy approved by the Chinese government after the implementation of the new type of urbanization strategy, which aims to establish a “super-integrated-region” across multiple regions as a benchmark to promote inter-provincial integration and help China achieve a unified national market. The study of Otsuka [26] supports Japan’s cross-regional national land planning policy, yet the empirical data from developing countries, such as China, are still not available. This study provides empirical support for the effectiveness of the government’s cross-regional integration policy. 

The rest of this article is as follows: Section 2 is the literature review and theoretical background of this study, Section 3 introduces data and methodology, Section 4 elaborates the results, Section 5 is the discussion, and the last section is the conclusion and recommendations. 

## 2. Framework for Understanding Regional Integration from the Perspective of Agglomeration and Urban Networks

### 2.1. Literature Review

The spatial influence of regional integration is one of the current focuses of global researchers. However, more attention is often paid to the interaction between regional integration and specific issues, such as welfare, income, politics, culture, and ecology [20,21,27,28,29,30]. Nevertheless, the regional integration process itself and its spatial consequences still remain poorly understood [31]. In the literature on regional integration, different views on regional integration process and its spatial consequences are expressed. First, empirical studies through economic growth channels argue that the process of regional integration has a positive spatial effect. For example, based on traditional agglomeration theory, Ke and Feser [8] as well as Shi et al. [14] believe that in an integrated group or big city (or center city) can obtain upgraded space by transferring redundant resources, while surrounding cities take on spillover industries and populations from the big city; this way, every city can leverage its comparative advantage to gain growth opportunities [10,13]. Additionally, Capello [7] and Camagni et al. [9] add that good transportation, communication and the division of labor may help to establish an interconnected urban network, in which each city can obtain the economic factors needed for its own development through complementary and cooperative methods to improve production efficiency. Finally, each city performs their own functions and achieves the effect of the overall regional income becoming greater than the sum of the income of a single city [11,12]. However, other researchers hold opposing viewpoints in accordance with the new economic geography “core-edge” model [6], which pointed out that in the regional integration practice, as the core area has a strong attraction for the peripheral area, population, capital and other economic factors will continue to gather in the core area when the regional internal accessibility increases, accompanied by an “agglomeration shadow”; a phenomenon that restricts the development of surrounding cities [6,32,33], where, in a specific region, the expansion of the core area comes at the expense of peripheral areas. Furthermore, once the circulation effect of the agglomeration works, the gap between core areas and peripheral areas will continue to widen. Some empirical studies have already verified the phenomenon of “agglomeration shadow” in the development of regional integration in China, the United States and ancient Europe [15,16,34]. Obviously, the possible growing gap between cities goes against the principle of regional integration to establish an interest community. On the other hand, the empirical research from developed countries based on the urban network theory (see Capello, 2000) mainly considers that, despite the economic correlation between most cities [35], whether the city integrates into the network and the depth of said network is positively related to the strength of the functional coupling between the city and the others [18]. Although the empirical study by Camagni et al. [9] and Otsuka [26] demonstrates that the peripheral areas can improve the production efficiency of achieving the ability of surpassing development without the need for more agglomeration costs by “borrowing” the technology, personnel and other factors of the production of the core areas. Additionally, Burger and Meijers [36] also disagree that urban networks form an “agglomeration shadow” that suppresses the growth of some cities because of a rat race among cities.

The above analysis shows that although the regional integration process and its spatial consequences are uncertain, its impact on the efficiency of overall regional and the equal development of urban individuals is clearly beyond doubt, especially for developing countries with low overall efficiency and large regional gaps. Clarifying its true development scenario and its possible spatial consequences may provide evidence for understanding the effectiveness of regional integration, which is the purpose of this study.

### 2.2. Theoretical Background

#### 2.2.1. Theoretical Framework

Urban agglomeration is essentially united by multiple cities with different degrees of connectivity. In this context, members share resources and other elements, thereby forming a relationship marked by both competition and cooperation. Meanwhile, urban agglomeration acts as a unified system to exchange materials and energy with outside areas [37]. Therefore, these areas should capture the essence of the system theory to achieve the ideal status of 1 + 1 > 2 [38,39]; that is, an urban agglomeration’s overall strength should be greater than the sum of its parts in order to pursue an urban community based on shared interests. This approach helps us form a working definition of urban agglomeration based on our scope.

The principle behind the metal valence bond theory is that atoms comprising a metal are interconnected by sharing electrons that are distributed on covalent bonds, and these covalent bonds are the sources of strength between connecting atoms. The longer the valence bond, the easier it is to break the inter-atomic linkage through external interference, thus contributing to weaker relationships between atoms and vice versa. Simultaneously, free electrons within the metal can participate in external exchanges when attracted to or interfered with by the perturbed system. Changes in temperature or other conditions bring about a dynamic interaction relationship in the system, forming different matching modes under different circumstances [40,41]. Accordingly, researchers have found that the flow characteristics of elements in urban agglomeration is consistent with the nature of electron exchanges in metals. Urban agglomeration is essentially similar to compounds formed by multiple elements (cities) through covalent bonds (cooperation), in which elements are shared by the internal members of a single urban agglomeration; meanwhile, the urban agglomeration and its peripheral system exchange substance and energy [42]. Here, the flow of elements may be subject to external disturbances, either actively or passively. Therefore, the metal valence bond theory provides a useful analogy for exploring interactions in city systems, both internally and externally. Likewise, cities form different dynamic and interactive relationships via element allocation, as the level of exchange shows the intensity of interaction. Under the influence of variable demands or capabilities, cities combine to form various matching modes.

#### 2.2.2. Conception: Coupling Distance

Based on the above theories, we defined “coupling distance” as the connection intensity between cities based on elements exchanged in material spaces; thus, a shorter coupling distance indicates a stronger interaction, while a longer coupling distance may eliminate the connection or coupling state. Here, the phenomenon of unbalanced development may appear based on one or both of the following conditions:

1. Passive state: if a peripheral city’s ability to offer comparative advantages and receive elements from the central city gradually weakens or competition with other cities pushes it out of the arena, it then becomes an “agglomeration shadow” within the urban agglomeration.
2. Active state: occurs when either the sending system (i.e., the peripheral city) actively seeks external contact and can thus divert its attention from the original core or the original center loses its centrality, thereby removing a member from the group. In both scenarios, the peripheral city becomes an “enclave” within the urban agglomeration.

**Figure 1:**
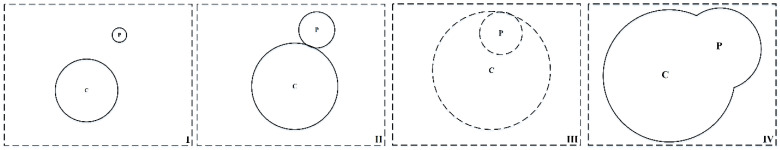
Model showing the course of integration for urban agglomeration (stages I–IV).

Both these conditions may enable cities to freely cooperate and combine efforts, which can move the boundaries of the urban agglomeration into a flexible range. In this context, we constructed a dynamic coupling relation model of integration (Figure 1) and developed corresponding formulas to identify the urban agglomeration as a proper scale and boundary. This process allowed us to assess both the integration process of the urban agglomeration and performance of the core city, then analyze the degree of the balanced development within the urban agglomeration.

In Figure 1, stage I represents a weak connection between the center city (C) and peripheral city (P), thereby resulting in separate entities. With a further connection, the strength of interaction becomes sufficient for balancing the power of interference, and the two cities reach critical integration, represented by stage II. Thus, there is no longer an inherent barrier between the central city and peripheral city, allowing an unimpeded access across elements and forming the complete integration area represented by stage III. In stage IV, the peripheral city gradually gains an ability to attract the central city into a state of further integration; as such, the two cities unite to form a newer and larger center. Notably, during a citywide recession, stages I–IV are reversible. Meanwhile, a “leap forward” in integration may occur; for example, stage II may result in an abrupt leap to stage IV. In general, however, urban agglomeration follows a course of integration that successively moves through.

## 3. Study Area, Data and Methods

### 3.1. Research Setting: Urban Agglomeration in the Middle Reaches of the Yangtze River

**Figure 2:**
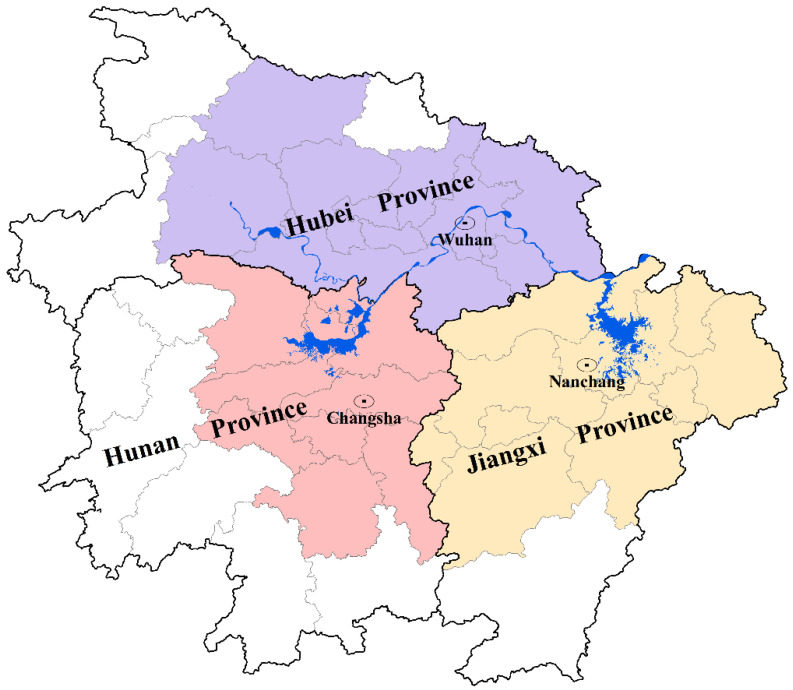
Urban agglomeration in the middle reaches of the Yangtze River in central China.

We chose the urban agglomeration in the middle reaches of the Yangtze River in central China (Figure 2) as our research setting, comprising Wuhan (core city: Wuhan, Hubei Province), Changsha-Zhuzhou-Xiangtan (core city: Changsha, Hunan Province), and the Poyang Lake (core city: Nanchang, Jiangxi Province) urban agglomeration. Together, they encompass 31 cities, with a surface area of 326,100 km^2^, accounting for 3.4% of the national land area. These areas had approximately 175 million inhabitants in 2019. 

The special inter-provincial grouping pattern exhibited by our research setting has been directed by government planning. In 2015, the Chinese government officially approved development planning for the urban agglomeration along the middle reaches of the Yangtze River, thus defining the current scale. However, the largest integration of regional planning members and size has not yet delivered the maximum overall economic efficiency; in fact, the economic efficiency is far less than that of the urban agglomerations of the Yangtze River Delta (26 cities), the Pearl River Delta (14 cities), and even Beijing-Tianjin-Hebei (13 cities). What accounts for this? Is it that the real integration scale of the urban agglomeration in the middle reaches of the Yangtze River does not reach that of the above three urban agglomerations, or is it that the market interaction among inter-provincial cities does not support the policy idea of inter-provincial integration and thus responded negatively? The answer to this question is important for planners or decision makers to adjust the future development direction of the integration of urban agglomeration in the middle reaches of the Yangtze River. In addition, as the forerunner of China’s multi-province integration planning, exploring the real process and consequences of the integration of urban agglomeration in the middle reaches of the Yangtze River can offer a reference to the latecomers.

### 3.2. Data 

Since 2012, Hubei, Hunan, and Jiangxi Provinces have determined the nature of cross-provincial cooperation in the middle reaches of China’s Yangtze River area. As such, this study relied on statistics, basic geographic data, and survey data from all three provinces covering the period lasting from 2013–2019. We obtained GDP data from the 31 cities and prefectures using the China Urban Statistical Yearbook, the China Urban Construction Statistical Yearbook from 2014–2020, and the statistical yearbooks of the corresponding years in each city. Geographic data included information cartographic maps (1: 5,000,000), as obtained from the Resource and Environment Data Cloud Platform of the Chinese Academy of Sciences. The spatial distances are calculated by ArcGIS 10.2 based on the 1:1 million national basic geographic database (2019 edition) provided by the China Geographic Information Resources Directory Service System, and the traffic distances are calculated using Baidu map API. Similarly, we determined the linear road distance between two given cities using Baidu map path planning (note: the traffic data used in 2013 was the same as that in 2019). Finally, the Baidu search index provided information on the strength of existing social connections from 2013–2019 [43].

### 3.3. Model and Measures

Our literature review revealed the importance of gravity models when assessing intercity economic linkages. The classical gravity model and its derivation use both the population number and gross product amount to express city strength. Taking the spatial distance between cities as obstacle factors, the central city’s force can be expressed in relation to its surrounding city. While this measure represents one-way economic linkages, it is inadequate for depicting the integration of an urban agglomeration conceptualized based on the “community of interests” concept. Considering the advantages/disadvantages of the gravity model and similarities between urban agglomeration and the metal valence bond theory mentioned earlier, the polyatomic interaction formula is appropriate for exploring the relationships between the constituents of the same urban agglomeration [44]. More specifically, this expression can help simulate the dynamic interactive relationship involving multiple atoms comprising solid compounds under temperature change, which applies to the spatial interaction variety of the urban agglomeration affected by intercity competition and cooperation. Within this framework, this study considered key influencing factors, including population, economic strength, traffic distance, and economic contribution rate, which have all been verified through previous empirical studies [45,46,47,48]. The fixed coefficient *β* in the original expression was improved to a dynamic $\beta_{s}$, which accurately describes the various potential connection strengths between different peripheral cities and the central city from the geographical perspective. We describe intercity economic relations using a quantitative formula adapted from the gravity model [49,50]. Combined with sociocultural factors within the network society, the coupling relationship between peripheral cities and the central city is expressed as follows:

(1) $$C_{p_{s}}=2D_{s}-\beta_{s}\log M_{s}$$

(2) $$\beta_{s}=k_{s}\frac{\sqrt{PV}\sqrt{P_{s}V_{s}}}{D_{t}^{2}}\;(k_{s}=\frac{V_{s}}{V+V_{s}})$$

In Formula (1), $C_{p_{s}}$ is the coupling distance between *s^th^* (peripheral city and central city). The coupling distance indicates whether the strength of the socioeconomic connection between the peripheral city and central city can offset material obstacles, including terrain and distance, and then achieve integration, and Table 1 shows the specific grading standards. $D_{s}$ is the distance from the *s^th^* city to the central city, while $\beta_{s}$ describes the strength coefficient of the intercity economic connection between the *s^th^* city and central city, and $M_{s}\;$ represents the strength of the social connection between the *s^th^* city and central city.

In Formula (2), $k_{s}$ is the contribution rate of the *s^th^* city to the intensity of intercity economic ties, while P and $P_{s}$ represent the permanent residents at the end of the year in the central city and the *s^th^* city, respectively. V and $V_{s}$ represent the annual GDP in the central city and the *s^th^* city, respectively, while $D_{t}$ is the shortest highway distance between the *s^th^* city and central city.

## 4. Results

### 4.1. Response of Spatial Hindering to the Range of the Urban Agglomeration

**Figure 3:**
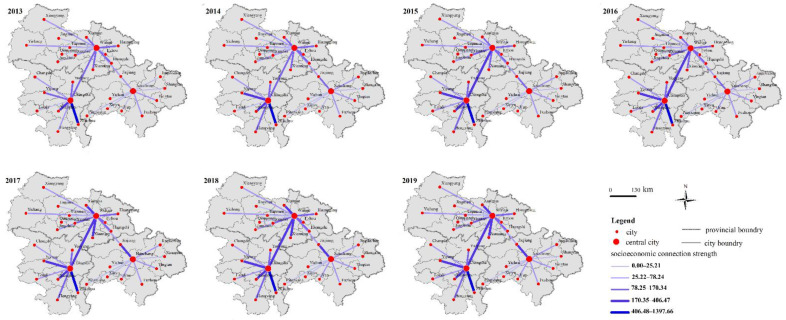
The strength of socioeconomic connections between peripheral cities and central cities (2013–2019).

Based on the 2013–2019 data, Figure 3 shows that the corresponding connection strength of peripheral cities is significantly different at different center distances; additionally, these differences have widened despite an overall strength increase. More specifically, the closer the city is to its central city, the higher is the socioeconomic connection between these cities within a 100 km range, which conforms to the distance attenuation law [51]. When cities are at distances ranging from 100–200 km from their centers, the corresponding intensity of the socioeconomic connection no longer presents a regular attenuation change, with a nadir even appearing at 150 km both in Wuhan and Changsha-Zhuzhou-Xiangtan urban agglomeration. Evidently, the low intensity of socioeconomic connections within distances ranging from 200–300 km illustrates an alienated relationship between the central city and its peripheral cities. However, Changsha and Nanchang, which are almost equidistant from Wuhan, perform differently. Figure 3 specifically shows that the socioeconomic connection between Wuhan and Changsha is the most outstanding among all cities, except Zhuzhou and Xiangtan from 2013–2019, nearly independent of the distance, but Nanchang’s response to Wuhan was indifferent. The strong connection between Wuhan and Changsha may encourage the cities to break spatial barriers and build a dual-core urban agglomeration in central China. 

Additionally, we noted that the space–time change of the strength of socioeconomic connections is also significant across the three sub-urban agglomerations. First, the socioeconomic connections within the Wuhan urban agglomeration and Changsha-Zhuzhou-Xiangtan urban agglomeration were both continuously strengthened, but Poyang Lake urban agglomeration showed a low-level balance state. Second, the magnitude of the former two socioeconomic connections was significantly higher than of the latter. However, despite the overall differences, they also have something in common. Therefore, we further sorted out the information presented in Figure 3 and analyzed the response of spatial blocking to the boundaries to help identify whether the cities within the three urban agglomerations were in a passive or an active state.

In the Wuhan urban agglomeration, the strength of the socioeconomic connection presents “a cliff-like drop” in Qianjiang and Tianmen, and both were approximately 150 km away from Wuhan. That is, Qianjiang and Tianmen are the lowest points of the whole Wuhan urban agglomeration, except for Jingmen, which is more than 200 km away. Regarding Changsha-Zhuzhou-Xiangtan and Poyang Lake urban agglomeration, to our surprise, the agglomeration shadows with the lowest linkage strength appeared at nearly 140 km and 150 km, respectively—for instance, Loudi (139 km) in Changsha-Zhuzhou-Xiangtan urban agglomeration and Xinyu in Poyang Lake urban agglomeration.

**Figure 4:**
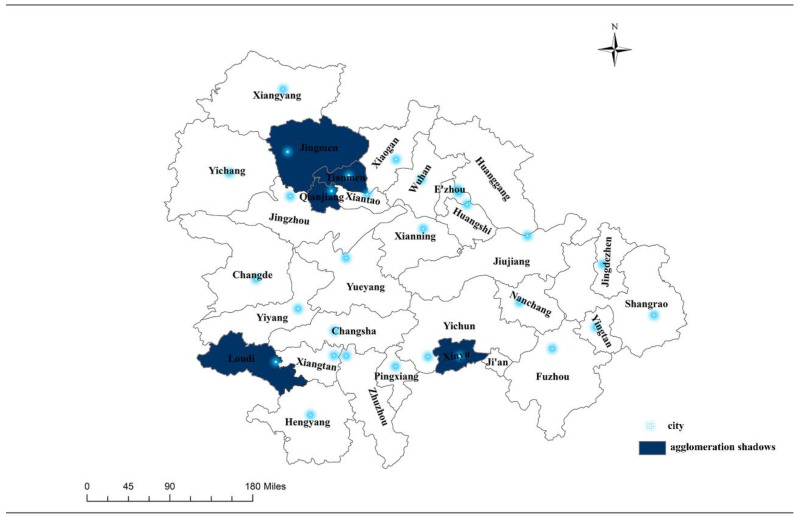
“Agglomeration shadows” in urban agglomeration in the middle reaches of the Yangtze River.

Based on the above reasoning, we drew the “agglomeration shadows” within the three sub-urban agglomerations (see Figure 4). As shown below, concerning socioeconomic connection strength, Qianjiang, Tianmen, Jingmen, Loudi, and Xinyu were the weakest among their clusters. By comparing these results with Figure 2, these five cities are almost all in the geometric center of their respective provinces. Thus, the findings showed a “geometric center collapse” in each secondary urban agglomeration; but their economic strengths were not the weakest nor were their spatial distances the farthest. 

### 4.2. State of Integration and Concrete Members of the Urban Agglomeration

Considering the separate states of the three secondary urban agglomeration shown in Figure 4, we explored the course of integration through concrete members from each urban agglomeration to further clarify the status quo of balanced development. Thus, we gained a perspective in which we see the superordinate urban agglomeration, allowing us to assess and solve potential problems in urban planning. 

**Figure 5:**
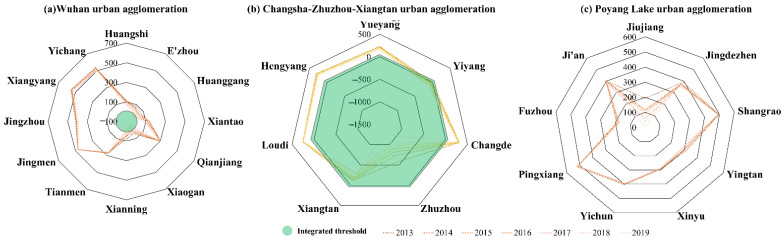
Evolution of coupling distance in three second-tier urban agglomerations (2013–2019).

As shown in Figure 5a, no city was integrated with Wuhan between 2013 and 2016. In 2017, Xiaogan was the first city to break the logjam; subsequently, Xianning and Huanggang joined the integrated nascent community in 2018 and 2019, respectively. Generally, these cities had a unidirectional attraction relationship with Wuhan. Moreover, our analysis showed that when cities within a 100 km distance from Wuhan accelerated towards integration, the other cities were relatively slow. In sum, peripheral cities exhibited significant differences during the integration process from 2013–2019, whereas the Wuhan urban agglomeration formed a substantial “community of shared interests,” comprising Wuhan, Xiaogan, Xianning, and Huanggang. This integrated group was far smaller than the nine cities suggested by a previous thesis focused on the Wuhan urban agglomeration, which included Tianmen and Qianjiang [52]. However, another study concluded that the Wuhan urban agglomeration was comprised Wuhan, Huangshi, and Xiaogan, thus implying that Qianjiang and Tianmen contributed little to the integration process [53].

As shown in Figure 5b, Zhuzhou, Xiangtan, and Yiyang have become substantially integrated within the Changsha-Zhuzhou-Xiangtan urban agglomeration since 2013; Yiyang and Zhuzhou, respectively, had a unidirectional and bidirectional attraction relationship with Changsha in 2013; Xiangtan achieved the same goal in 2017. However, in the Changsha-Zhuzhou-Xiangtan urban agglomeration, the peripheral and central cities exhibited a polarized integration process. Conversely, cities adjacent to Changsha were rapidly joined with Changsha from 2013–2019, resulting in a high level of integration; indeed, Changsha, Zhuzhou, Xiangtan, and Yiyang combined to form a community of shared interests. In contrast, the remaining peripheral cities showed little change from 2013–2019. This result was distinctly different from that of an earlier study based on intercity bus frequency data, which argued that Changsha, Zhuzhou, Xiangtan, Changde, and Hengyang constituted the Changsha-Zhuzhou-Xiangtan urban agglomeration [54]. In contrast, our results are supported by a recent study based on population, GDP, and road network data, which concluded that Changsha, Zhuzhou, and Xiangtan had achieved integration from a transportation standpoint [55]. 

Figure 5c shows a highly nascent integration of the Poyang Lake urban agglomeration during 2013–2019, with little changes in regional coupling distance except in Jiujiang. Fuzhou and Pingxiang even exhibited an anti-coupling trend in recent years, otherwise known as a “retreat group.” This result is contrary to that of a previous study suggesting that the economic hinterland of the Poyang Lake urban agglomeration included Nanchang, Jingdezhen, Fuzhou, and Shangrao [56]. However, while most related studies have shown that this region did not truly form an urban agglomeration [40,57,58], none of those researchers were aware of the anti-coupling phenomenon at the time of investigation. Moreover, further analysis considering the anti-coupling trend of Pingxiang and its proximity to the Changsha-Zhuzhou-Xiangtan urban agglomeration showed that Pingxiang-Changsha has a higher degree of connection than Pingxiang-Nanchang. That is, Pingxiang seems to be an enclave in the Poyang Lake urban agglomeration.

**Figure 6:**
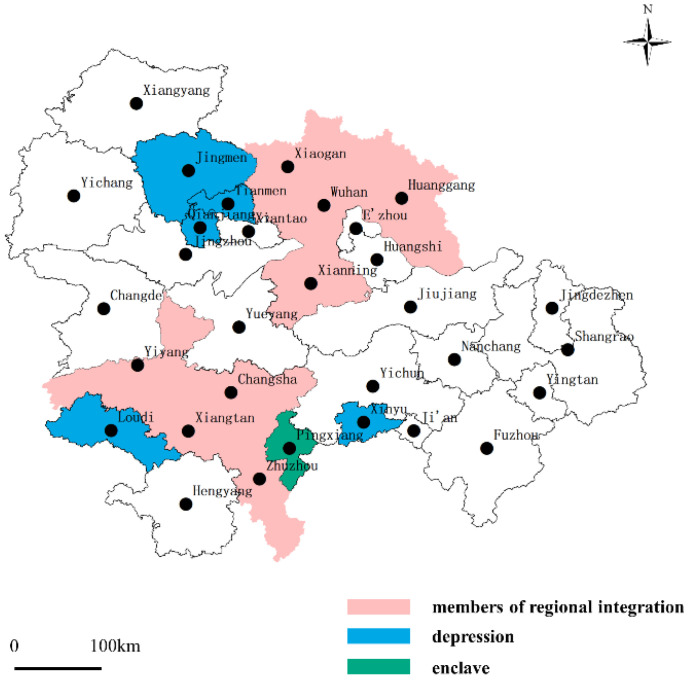
Integration conditions for the urban agglomeration in the middle reaches of the Yangtze River in 2019.

By integrating the above analysis, we see concrete members forming the urban agglomeration in the middle reaches of the Yangtze River (Figure 6). In 2019, the Wuhan urban agglomeration comprised Wuhan, Xiaogan, Huanggang, and Xianning; thus, the actual membership number was less than one quarter of that outlined in the original planning stage. Meanwhile, the Changsha-Zhuzhou-Xiangtan urban agglomeration included Changsha, Zhuzhou, Xiangtan, and Yiyang, accounting for half of that outlined in the original planning. In contrast, there was no urban agglomeration around Poyang Lake. In sum, the urban agglomeration in the middle reaches of the Yangtze River comprises eight cities within two separate urban agglomeration, constituting one quarter of what was planned. In addition, Tianmen, Qianjiang, and Jingmen are agglomeration shadows in the Wuhan urban agglomeration, similar to those that Loudi formed in the Changsha-Zhuzhou-Xiangtan urban agglomeration, and Xinyu formed in the Poyang Lake urban agglomeration. Meanwhile, Pingxiang appeared as an enclave that had left the Poyang Lake urban agglomeration.

## 5. Discussion 

Our study findings are partly consistent with previous studies. For example, we believe most cities in the urban agglomerations in the middle reaches of the Yangtze River yet have not formed urban networks that are free from spatial constraints at this stage. Additionally, regional integration is more susceptible to localization economies at the provincial level. This view echoes the observations of Duranton [19] and Glaeser et al. [17]. They also believe that the importance of urban networks is not significant for small and medium-sized cities in developing countries or underdeveloped areas. Furthermore, compared with the standard method that converts the localization socio-economic behaviors into network connections stripped of spatial properties, our conclusions prove that examining network connections in the local spatial dimension applies even more in developing countries. Over-belief in urban networks without strict space restrictions will probably mislead developing countries to draw a large scale in planning regional integration, resulting in negative consequences. 

The method of examining network connections in the local spatial dimension proposed in this study regards spatial constraints as the obstacles that must first be broken through in developing regions, although we acknowledge the role of spatial constraints and urban networks in integration. Removing spatial constraints means that the economic, social, and traffic links between the two cities reach a high level, likely to further form a city network that is not subject to spatial constraints. Therefore, our method reveals the multi-stage leading factors corresponding to the integrated development of urban agglomeration.

Second, we provide empirical evidence to support the “agglomeration shadow” phenomenon, a finding that is in line with previous theoretical literature [6,7,33] and some empirical studies [15,16,34,36], which proves that the “coupling distance” model proposed in this paper is scientific and reasonable. However, these studies also differ from the idea proposed by Bosker and Buringh [15] that the farther away from the center city (within, 136–399 km), the more pronounced the agglomeration shadow effect. This study found that the agglomeration shadow appeared at locations about 140–150 km away from the center cities. Other cities outside this range have more positive interactions with their center cities. Customarily, a city at a geometric center is assumed to have more geographic neighbors, with a higher degree of convenience in communicating with the outside world. However, our empirical analysis showed that their performance is reduced to an “agglomeration shadow.” Are too many choices distracting them? Or are the neighbors blocking their connection with the central city? Or could it be related to the spatial structure of the integrated region [17]? The empirical analysis shows what seems to be a “center position curse” despite these reasons. Is this phenomenon common in other government-led regional integration countries? We speculate that this curse may appear regularly in rapidly urbanizing areas and propose the “central position curse hypothesis” in a nutshell. We will not go into the details here but will follow up with more empirical tests on this hypothesis in future studies.

In addition to the above findings, which are consistent with previous studies, the phenomenon of “inverse integration” and “enclave” in this study are new findings. First, the nature of the “inverse integration” phenomenon differs from that of the “agglomeration shadow.” The peripheral cities where “inverse integration” occurs are not those whose growth is suppressed by the center city but those that run counter to the integration. It may be a widespread phenomenon in regional integration planning in many developing countries. This is because the “1 + 1 > 2” effect is the motivation that propels many countries to implement regional integration policies. However, people may mistake this agglomeration effect as “more member cities will produce more benefits.”. For example, in China, planners often try to include most cities in a province when deciding the scale of an integrated region, but this often fails to achieve the desired overall benefits and ultimately leads to an ineffective spatial agglomeration strategy of “large but weak.” Similarly, the Moscow–St. Petersburg megalopolis, the megacity of Mumbai, and Mexico City also have the same problems [59,60,61]. Second, the phenomenon of the “enclave” in China may be related to the conflict between China’s “centralization–local decentralization” administrative system and the goal of regional integration. More specifically, China’s regional integration is implemented by way of “local application–central government approval.” China’s central government wants to break local protectionism to achieve the goal of cross-province integration. However, the provincial government is trying to reduce the administrative costs and the management conflict with other governments at the same level. Hence, as the premise of cities outside their administrative area are not generally considered, resulting in the misplaced integration with market behavior counter to government behavior. Of course, a strong central city and a lively peripheral city are the primary conditions for this situation. Therefore, whether this phenomenon is an exceptional case or whether it commonly appears in the multi-center or cross-regional integration process remains to be further explored. Generally, both above phenomena signal the readjustment to government-led regional integration planning. For most developing countries dominated by government intervention, this study can help government officials carry out the more effective planning and restructuring of regional integration.

## 6. Conclusions

In the present study, we revealed the extent of regional integration and its spatial consequences in the urban agglomerations in the middle reaches of the Yangtze River by applying the “coupling distance” model. There are three findings regarding the regional integration process of these urban agglomerations. First, on a local scale, the integration process of secondary urban agglomeration shows noticeable trend of local gathering. Moreover, the intensity of the network ties between cities is subject to strict physical space constraints. In contrast, on a macro-scale, the connections between central cities break free from distance and division, showing the positive effect of the urban network on cross-regional integration. Finally, the actual integration range of the whole urban agglomeration in the middle reaches of the Yangtze River is far smaller than its planning scale, which implies that the market-led regional integration process lags behind the original assumptions of the government, resulting in some significant spatial consequences. 

Specifically, this study verifies that the “agglomeration shadow” phenomenon appears in urban agglomerations in the middle reaches of the Yangtze River. A more significant concern is that peripheral cities in the shadows are 140–150 km away from their central cities. In contrast, the sizes of the economies of three secondary urban agglomerations and three central cities (Wuhan, Changsha and Nanchang) are significantly different. Moreover, the above peripheral cities in the agglomeration shadows are all located at the geometric geographical center of the province with net population outflow (the population size decreases). Second, this study found that the “inverse integration” phenomenon in the Poyang Lake urban agglomeration planned by the government and the coupling effect among several peripheral and central cities is weakening. Similarly, the “enclave” phenomenon has also occurred in the Poyang Lake urban agglomeration. The integrated coupling degree between its peripheral city (i.e., Pingxiang) and the closer cross-regional central city (Changsha) is much higher than that between Pingxiang and the prescribed central city (Nanchang).

Our findings have a substantial value in theory and policy application. Theoretically, although urban networks have emerged in developing countries, regional network sharing is still subject to distance and division. In contrast, most recent studies from developed countries hold an excessively positive attitude toward urban networks replacing traditional agglomeration. It is necessary to reconsider the appropriateness of an “urban network”, whether it is a virtual network outside of geographic space or a local network of a geographically embedded nature. For developing countries, completely ignoring the impact of locality on urban networks may bring too large a spatial scale to obtain the desired agglomeration effect in regional integration planning. Indeed, the excellent network connections between central cities affirm the effectiveness of the Chinese government’s cross-regional integration policy. In addition, the different performances of an urban network in the same region in China also corroborate the findings of previous studies that the spatial impact of regional integration is uncertain. This study suggests that this spatial impact should be periodic rather than uncertain.

Given the constraints of the article length and the need to focus on the theme, our study has certain limitations. Future studies should deepen the thinking of the above conclusions from the perspective of genesis, mechanism, etc. For example, in addition to presenting the phenomena, we should also explore why urban networks are subject to geospatial factors, such as distance, and the fundamental mechanisms of the phenomenon of “inverse integration” and “enclave.” Meanwhile, as for the agglomeration shadow, the range is theoretically different due to the differences in size and development conditions of various cities. In the study, the agglomeration shadows of the three secondary urban agglomerations are surprisingly consistent with the distance and the relative geographical location of their respective central cities. This has not yet been studied in depth. In particular, it is necessary to clarify why agglomeration shadows appear in provincial geometric geographical centers. Moreover, the “coupling distance” model proposed in this study used the search index of China’s local search engines (i.e., the Baidu Index) to represent the social (information) ties between cities. Google trends can be used as an alternative to improve the model’s applicability in other countries or regions.

## Figures and Tables

**Table 1:** Division of key nodes in the integrated development of urban agglomerations.

Classification	Integrated Response Status	Meaning
0 < $C_{p_{s}}$ ≤ 2D_s_	weak response	the peripheral cities and the central cities have weak connections or even have no connection;
$C_{p_{s}}$ = 0	integrated initial response	the strength of interconnection between peripheral cities and central cities initially offsets the spatial interference;
∣$C_{p_{s}}$∣ ≥ 2D_s_, $C_{p_{s}}$ < 0	fully responsive integration	peripheral cities and central cities completely overcome all interference and obstacles and they enter complete integration;
∣$C_{p_{s}}$∣ ≥ 4D_s_, $C_{p_{s}}$ < 0	bidirectional response	the peripheral cities have the ability to attract the central cities in a reverse direction, and they finally form a community of interests.

## Data Availability

The data that support the findings of this study are available from the corresponding author, upon reasonable request.
